# Device implant based on poly (lactic acid) with vitamin E for vaccine delivery system in Tilapia: Study for biocompatibility and biodegradation

**DOI:** 10.1016/j.fsirep.2022.100060

**Published:** 2022-07-03

**Authors:** Gabriel Conde, Mayumi Fernanda Aracati, Letícia Franchin Rodrigues, Susana Luporini de Oliveira, Camila Carlino da Costa, Ives Charlie-Silva, Thalles Fernando Rocha Ruiz, Sebastião Roberto Taboga, Marco Antonio de Andrade Belo

**Affiliations:** a Department of Preventive Veterinary Medicine, São Paulo State University (Unesp), Rodovia de Acesso Paulo Donato Castellane s/n, Zona Rural, zipcode 14884-900, Jaboticabal, SP, Brazil; b Deparment of Pharmacology, Institute of Biomedical Science, University of São Paulo (USP). 2415 Prof. Lineu Prestes Avenue, Cidade Universitária, São Paulo SP, ZipCode:05508-000, Brazil; c Laboratory of Animal Pharmacology and Toxicology, Brazil University, Hilário da Silva Passos avenue, 950, CEP.13690-000, Descalvado, SP, Brazil; d Department of Biology, São Paulo State University (Unesp), 2265 Cristóvão Colombo, Jardim Nazareth, zipcode 15054-000, São José do Rio Preto, SP, Brazil

**Keywords:** Biomaterials, Oreochromis niloticus, Clinical safety, Inflammatory response, Innate immunity

## Abstract

The use of Poly (lactic acid) (PLA) as a slow-release vehicle for vaccines has attracted the attention of researchers, since its insertion improves the uptake of them, and reduces side effects or by stimulating recruited defense cells, assisting immunity without the need for booster vaccine doses. Seeking to develop new strategies for the administration of drugs and vaccines in aquaculture, we evaluated the biocompatibility and biodegradation of polymeric PLA devices and PLA plus vitamin E devices, implanted through subcutaneous (SC) and intraperitoneal (IP) routes in Nile tilapia. To carry out this study, 84 male tilapia (initial 243.82 ± 56.74 g; final 400.71 ± 100.54 g) were randomly distributed in 3 tanks (*n* = 28 fish per treatment/tank). The devices were prepared in two formulations: neat PLA (containing 100% PLA) and PLAVE (PLA plus vitamin E) implanted using a commercial AnimalTag® applicator, and non-implanted fish (control). Fish were sampled 15, 30, 60, and 120 days post-implantation (DPI). Blood analysis was used to access blood cells and blood smear for differential leucocytes count. Serum biochemistry to evaluated changes in serum proteins and glycemia. Histopathological investigation using hematoxylin-eosin (H&E) was used to assess polymer-tissue interaction. Histochemistry and immunohistochemistry was used to detection immune cells and phagocytes in capsule, and analyses of melanomacrophage centers (MMCs) to morphometric evaluation and percentage amount of melanin, hemosiderin and lipofucsin pigments. Histopathological study revealed an increase of capsular formation and inflammatory cell infiltration in PLAVE-implanted tilapia through SC route (15 DPI). Tilapia implanted with PLAVE and PLA (SC) presented mast cells and eosinophilic granular cells during 15, 30, and 60 DPI, with a decrease in these cells in the fibrous capsule around the polymer at 120 DPI. PLAVE implanted tilapia SC at 60 DPI showed significantly phagocytosis points than other groups. Phagocytic cells (F4/80+) were observed near to biopolymers in phagocytosis sites. Lipofuscin at 120 DPI in spleen melanomacrophage centers were significantly high in PLAVE implanted tilapias when compared to fish with PLA implants and control. The serum biochemical study of tilapia did not reveal changes in cytotoxicity and liver function in implanted fish. The absence of side effects in hematological and biochemical findings, including the absence of mortality after device implantation, proves its clinical safety. PLA implants in tilapia have demonstrated biocompatibility, biodegradation, clinical safety, and excellent evolution of foreign body inflammatory responses.

## Highlights

• PLAVE implants resulted in increased of capsular formation, phagocytosis, and cellular infiltration.
• PLAVE-implanted tilapia presented increase in melanin, hemosiderin, and lipofuscin in spleen MMCs.
• The absence of side effects in hematological and biochemical findings proves PLA's clinical safety.
• PLA implants in tilapia have demonstrated biocompatibility and biodegradation .

## Graphical abstract

**Figure:**
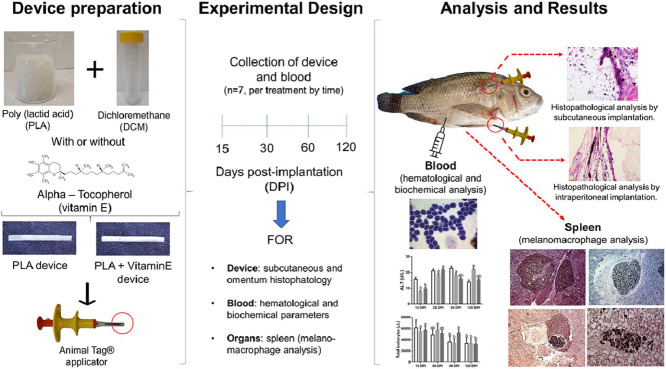

## 1 Introduction

The use of biomaterials from renewable sources has been growing in several biomedical segments, among them, the poly (lactic acid) (PLA) has shown great interest in scientific community, since it has been used as bone fixation material [1], coronary stents [2], tissue engineering [3], drug, and vaccine-carrying microspheres [4], [5], [6]. Other authors have studied the biocompatible and biodegradable of pure PLA and PLA with other polymers called blends and, whit another compounds, suggesting that the PLA is safety [7], [8], [9]. The PLA was approved by the FDA in 1971 for the development of sutures. After implantation the product of the biodegradation by phagocytosis results in the transformation of L-lactate monomer in pyruvate, which is a substrate for the Krebs cycle and oxidative phosphorylation, resulting in the production of carbon dioxide and water, and both are easily excreted by the body [10].

Vaccines with PLA-based vehicles may have advantages, as they result in prolonged inflammatory stimuli with the accumulation of defense cells recruited to the implant site [11], [12]. Franz et al. [13] described the implantation of PLA activates the pathogen-associated molecular patterns (PAMPs) and through macrophages, dendritic cells, and pattern recognition receptors (PRR) to promote inflammation. The long period of PLA degradation may keep the antibody titers elevated favoring the animal against opportunistic pathogens such as the case of Streptococcus agalactiae and Aeromonas hydrophila in tilapia farms, without the need for reinforcement vaccine doses, reducing handling stress, and production costs [4], [5], [6], [7], [8], [9], [10], [11], [12], [13], [14], [15], [16].

The preparation of polymers as vaccine carriers in mammals [8], [9], [10], [11], [12], [13], [14], [15], [16], [17] and fish [18] is carried out using oil-in-water (o / w) emulsion methodology and most commonly water-oil-water (w / o / w) in which the antigen or the active molecule is soluble in a non-water-miscible solvent, such as dichloromethane (DCM). A common step for both techniques is the dispersion of the polymer in an aqueous phase containing the emulsifier polyvinyl alcohol [4]. Researchers proposed the use of natural emulsifiers for the preparation of microspheres for the distribution of drugs, among them vitamin E [19;20]. The process of producing microspheres using vitamin E is easier to manufacture and is also more effective in the encapsulation process [20].

Schubert et al. [21] demonstrated that the use of vitamin E incorporated into poly (etherurethane urea) implanted subcutaneously in rats improves biocompatibility and stimulates a greater amount of macrophages adherent to the material during the inflammatory reaction. McNally and Anderson [22] observed that α-tocopherol promoted adhesion with cytoplasmic dissemination, favoring macrophage fusion and formation of foreign body giant cells (FBGC). These authors reported that α-tocopherol contributed to the formation of FBGC in the presence of interleukin-4 (IL-4), and also induced the formation of these giant cells through the activation of diacylglycerol kinase.

Furthermore, vitamin E has important antioxidant activity maintaining the flow of nutrients in phagocytes [23] and favoring the defense mechanisms of fish during the foreign body's chronic inflammatory reaction [24,25]. Vitamin E accumulation in tissues helped to improve immune regulatory response and defense against stressors and infectious pathogens in Nile tilapia by upregulated gene expression in an oral administration [26,27].

Based on the importance of developing new strategies for the administration of drugs and vaccines in aquaculture, we evaluated the biocompatibility (clinical safety) and biodegradation of neat polymeric poly (lactic acid) devices and poly (lactic acid) plus vitamin E, implanted through subcutaneous and intraperitoneal routes in Nile tilapia.

## 2 Materials and methods

### 2.1 Animals and experimental design

To carry out this study, 84 male tilapias (243.82 ± 56.74 g), from Aquabel farm (Porto Ferreira, São Paulo State, Brazil) and belonging to the same spawning, were randomly distributed in 3 tanks (1000 L of water, *n* = 28 fish per treatment/tank) with recirculation system at a flow rate of 5 L min^−1^, to perform the following three treatments: control group (without implant), PLA and PLAVE (PLA with Vitamin E). Fish were reared in this system for 3 months before starting the study with the implantation of biomaterials. Fish were fed 3% of biomass with commercial feed (Nutripiscis® - Neovia Company, 28% GP, and 4000 kcal of GE kg^−1^). Water quality parameters were determined daily using pHmeter with condutivimeter (model YSI-63) and oximeter (model YSI-55), and their values remained within the adequate range for tropical fish comfort [28] (dissolved oxygen = 4.07 ± 0.89 mg L^−1^: temperature =27.64 ± 2.05 °C; pH = 7.64 ± 0.54; and conductivity = 208.29 ± 97.57 μS/cm). This research was approved by the Ethics Committee for the Use of Animals belonging to São Paulo State University, FCAV-UNESP, protocol n° 08,665/19.

### 2.2 Device of poly (lactic acid) and implantation

The devices were prepared in two formulations: neat PLA containing 100% of poly (lactic acid) and PLAVE containing poly (lactic acid) plus vitamin E. Basically, 500 mg of poly (lactic acid) (PLA grade: Ingeo 3251D, manufactured by NatureWorks Co., Ltd) was dissolved in dichloromethane (DCM) stirring for 20 min. To produce PLAVE, the same methodology described was used, obtaining the dissolution of PLA, vitamin E was added to the dissolved PLA [20]. Thus, PLA or PLAVE were placed in glass capillaries maintained at room temperature during four days for drying. The devices were removed from the capillaries and sectioned with one centimeter of length (patent number BR 10 2020 026,197 5). Prior to implant, the devices were immersed in 70% ethanol for one hour and dried in an oven [29,30]. Then the fish were anesthetized by immersion in 1: 10.000 (v: v) aqueous solution of benzocaine (Sigma Chemical Co., St. Louis, Missouri 63,178, USA), and using a commercial AnimalTag® applicator, the same fish receive two devices of PLAVE or PLA implanted intraperitoneally (IP) and subcutaneously (SC).

### 2.3 Blood analysis

Seven fish per treatment per time (totalized *n* = 28 fish/treatment) were anesthetized (item 2.2) to obtain blood samples from the caudal vessel at 15, 30, 60, and 120 days post-implantation (DPI), using two sets of needle and syringe one coated with lithium heparin and another without anticoagulant to obtain plasma and serum samples, respectively. Blood cell counts were realized by hemocytometer (Neubauer chamber) and Natt and Herrick solution (proportion of 1:100 v:v). The hematocrit (Ht) was determined in microhematocrit centrifugation technique and hemoglobin concentration (Hb) with Drabkin´s reagent read at 540 nm. Mean corpuscular volume (MCV) and mean corpuscular hemoglobin concentration (MCHC) were calculated from the Ht, [Hb], and red blood cells. Blood smears for differential leukocyte counts were stained with a combination of May-Grünwald Giemsa and Wright´s Method [31]. After blood sampling, fish euthanasia was carried out by prolonged exposure to benzocaine hydroalcoholic solution 1:500 (v:v).

### 2.4 Reactive oxygen species (NBT assay)

The respiratory burst of leukocytes was measured according to Farias et al. [32]. For that, 100 μL of an NBT-buffered solution at 0,2% (NBT-nitroblue tetrazolium, Sigma, St. Louis, MO, USA) was mixed with 100 μL of heparinized blood. This solution was homogenized and incubated in a dark room for 30 min at 25 °C. After the incubation, 50 μL of the solution was added to 1 mL of n,n-dimethyl-formamide (DMF, Sigma, St. Louis, MO, USA), and centrifuged at 3000 g for 5 min. The supernatant optical density was measured using a spectrophotometer (Beckman DU-70S) with a wavelength of 540 nm.

### 2.5 Serum biochemistry

Fish blood samples without anticoagulant were centrifuged at 3000 g for 10 min. at 4 °C to obtain the serum for total protein, alkaline phosphatase (ALP), aspartate aminotransferase (AST), and alanine aminotransferase (ALT) determination, using in a semiautomatic biochemical analyzer (Model LabQuest® – Bioplus Company) [33] and fish glycemia was determined using the Accu-Chek Performa device.

### 2.6 Histopathology

The devices implanted in the SC were removed together with subcutaneous tissue and adjacent skeletal muscle, while the IP implants were collected with the omentum. Therefore, samples of polymeric implants and splenic tissue for melanomacrophage studies were fixed in 10% buffered formalin, embedded in paraffin, sectioned at 5 µm, and stained with hematoxylin and eosin (H&E) for photomicroscopic assessment (Carl Zeiss Jena, Germany) and images captured using software (Opton CMOS). An experienced pathologist performed blind histopathologic analyses and the histological findings related to polymeric implants were quantitatively classified using a numerical score: 1-mild reaction, 2-moderate reaction, 3-intense reaction, and 4-severe reaction according to the criteria proposed by De Jong et al. [34].

#### 2.6.1 Eosinophilic granular cells (EGCs) and phagocytosis points count

Histological sections were stained in H&E, for counting evaluation. For this purpose, five fields per animal were randomly selected and photographed (Opton CMOS TA-0124-D) totaling 35 fields per treatment in each evaluated period (15, 30, 60, and 120 DPI), to be determined the area (mm^2^) the number of eosinophilic granular cell, and phagocytosis points per field were counting using the Image-J program [35]. Number of cells or phagocytosis points were divided by area (cell or phagocytosis / mm^2^).

#### 2.6.2 Histochemistry and Immunohistochemistry

For detection of immune cells and phagocytes in capsule, histochemistry and immunohistochemistry (IHC) analysis were performed. The Periodic Acid Schiff (PAS) staining was performed to identify the leukocytes-granulocytes infiltrated in capsular tissue. Toluidine blue (1%, pH 4.0) staining was performed to identify mast cells in tissue sections through metachromatically reaction of heparin and sulfated glycosaminoglycan granules. For IHC detection of phagocytes, histological sections were submitted to reaction with the primary antibody F4/80 (rabbit monoclonal, 1:100, D2S9R, #70,076, Cell Signaling, Danvers, MA, USA). Slides were dewaxed, and antigens were retrieved in 10 mM citrate buffer (pH 6.0, at 98 °C). Endogen peroxidases blocked in 10% H_2_O_2_, and nonspecific proteins were blocked using 5% skimmed milk. After, they were incubated overnight with the primary antibodies. An incubation with polymer (Novolink Max Polymer DS (1250), Leica) was performed, and detection was performed with 3–30′-diaminobenzidine tetrahydrochloride solution (DAB) and counter-stained with Harris Hematoxylin, staining and inspected under a light microscope (Olympus BX50, Olympus Corporation, Center Valley, PA, USA). Images were captured using Olympus cellSens Standard 1.18 software (Olympus Corporation, Center Valley, PA, USA).

### 2.7 Analysis of melanomacrophage centers (MMCs)

Spleen histological sections were stained in H&E, toluidine blue, Perl's (hemosiderin - ferric blue pigment), and Schmorl's (lipofucsin - brown pigment) stain for morphometric evaluation and the percentage amount of melanin, hemosiderin, and lipofucsin pigments present in MMCs, following the methodology described by Manrique et al. [36]. For this purpose, five fields per animal were randomly selected and photographed (Opton CMOS TA-0124-D) for each staining, totaling 35 fields per treatment in each evaluated period (15, 30, 60, and 120 DPI), to determined the area (μm^2^) and the number of MMCs per field, as well as the percentage of each pigment, using the Image-J program [35].

### 2.8 Statistical analysis

The data were tested for normality using the Kolmogorov-Smirnov test. Data were analyzed using a linear model, which included fixed effects for treatment and treatment-time to implant interaction. Differences between treatments were determined by the Kruskal-Wallis test. All analyzes were carried out using Sigma-Plot, version 12.0. Significant differences (*p* < 0.05) were estimated based on Dunn's test.

## 3 Results

### 3.1 Polymeric implants

**Fig. 1:**
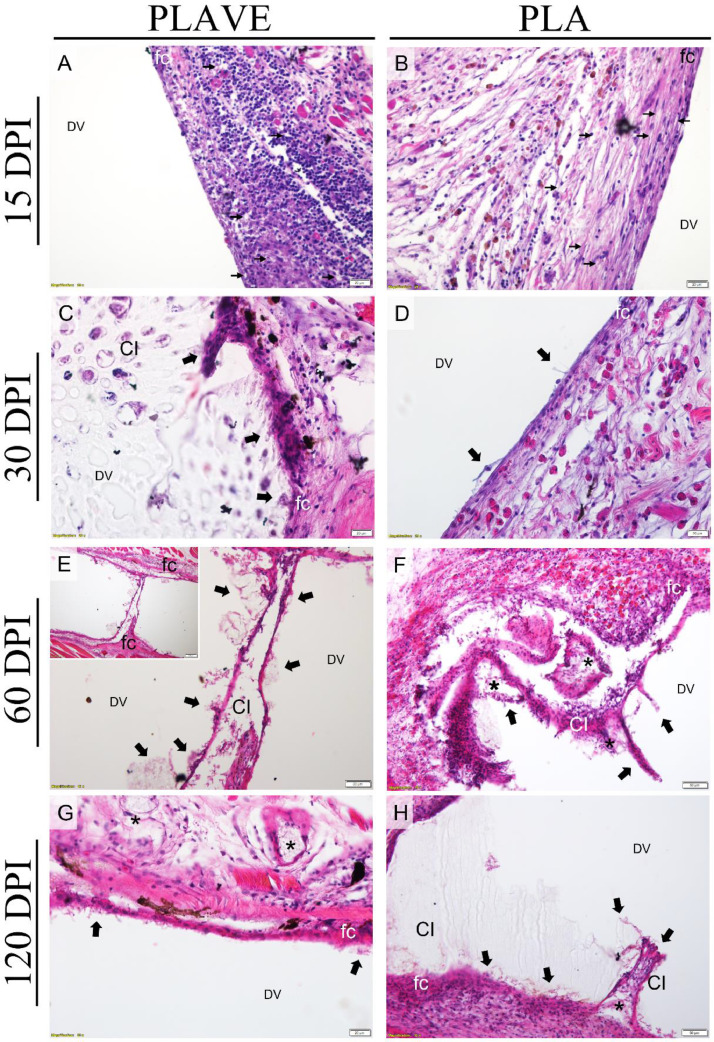
Photomicrographs of PLAVE and PLA subcutaneous implants in Nile tilapia (Oreochromis niloticus), stained with H&E. (A - B) diffuse mononuclear inflammatory infiltrate in the skin tissue adjacent to the fibrotic capsule (fc), neovascularization in the capsule and skin tissue (thin arrows), and in (B) presence of the eosinophilic granular cell; (DV) polymer location region (scale bar = 20 μm). (C - D) fibrotic capsule (fc) with phagocyte cells paving and tissue projections on the polymer (thick black arrow), and in (D) presence of the eosinophilic granular cell in capsule; (CI) cell infiltration within the polymer, (DV) region of polymer location (scale bar = 20 μm). (E - F) fibrotic capsule (fc) with phagocyte cells paving and surrounding the polymer (thick black arrow), and (*) phagocytosis points, (CI) cell infiltration within the polymer, phagocytosis points (*), (DV) polymer localization region (scale bar = 50 μm). (E inset) cell bridge connected to fibrotic capsule (fc) between the ends of the polymer (scale bar = 100 μm) (G - H) fibrotic capsule (fc) with macrophage paving and tissue projections on the polymer (thick black arrow), multinucleated giant cells and phagocytosis points (*), (CI) cell growth within polymer, (DV) polymer location region (D; scale bar = 20 μm; E; scale bar = 50 μm).

**Fig. 2:**
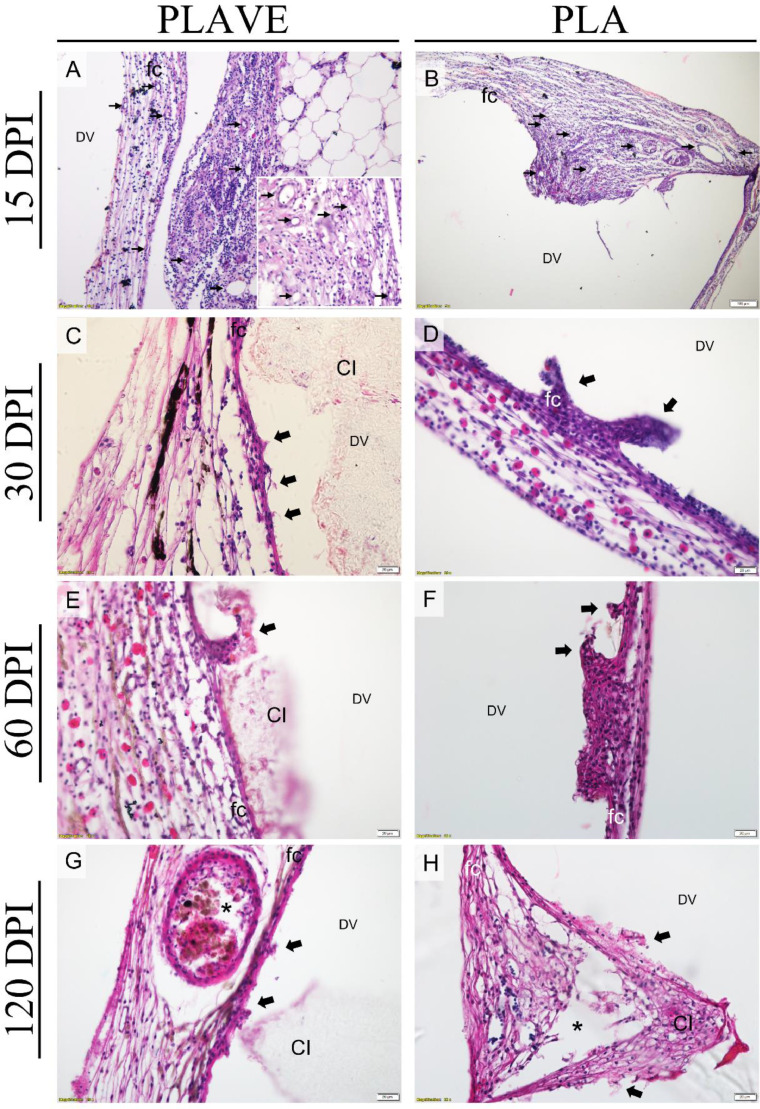
Photomicrographs of PLAVE and PLA intraperitoneal implants in Nile tilapia (Oreochromis niloticus), stained with H&E. (A - B) diffuse mononuclear inflammatory infiltrate in the omentum adjacent to fibrotic capsule (fc), neovascularization in the capsule and omentum (thin arrows), and (DV) polymer location region (A; scale bar = 50 μm; E; scale bar = 100 μm) (A inset) neovascularization (thin arrows) in omentum (bar = 20 μm). (C - D) fibrotic capsule (fc) with macrophage paving and tissue projections on the polymer (thick black arrow), and in (D) presence of the eosinophilic granular cell in capsule. (CI) cell infiltration into the polymer and (DV) region of polymer location (scale bar = 20 μm). (E - F) fibrotic capsule (fc) with macrophage paving and tissue projections with phagocyte cells into the polymer and surrounding the polymer (thick black arrow), and in (E) presence of the eosinophilic granular cell in capsule, (CI) cell infiltration within the polymer, and (DV) polymer location region (scale bar = 20 μm). (G - H) fibrotic capsule (fc) with macrophage paving and tissue projections on the polymer, phagocyte cells surrounding the polymer (thick black arrow), and (*) phagocytosis points, (CI) cell infiltration into the polymer and (DV) region of polymer location (scale bar = 20 μm).

The first assessment at 15 days post-implantation (DPI) was performed to verify the formation of a moderate capsule with macrophages between the implant and fibrous capsule. In the following evaluations, cytoplasmic projections were observed involving parts of the polymers containing paved macrophages adjacent to the fibrous capsule in subcutaneous (Fig. 1), and intraperitoneal implants (Fig. 2). Neovascularization in the fibrous capsule can be seen with greater intensity at 15 and 30 DPI. However, at 60 and 120 DPI, a notable increase in the diameter of blood vessels and retraction of the capsule was observed with the presence of focal points of phagocytosis and the presence of cells inside the polymer (Fig. 1 and Fig. 2).

**Fig. 3:**
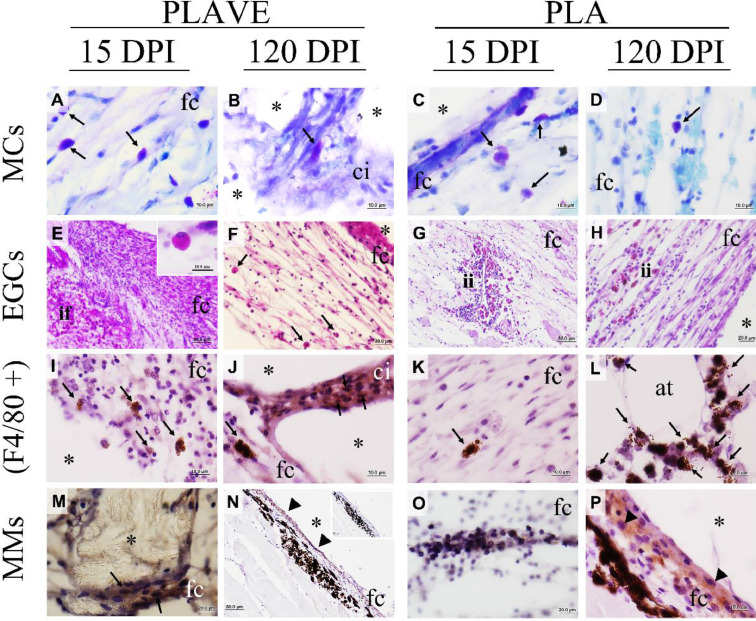
Photomicrographs of PLAVE and PLA intraperitoneal and subcutaneous implants in Nile tilapia (Oreochromis niloticus). (A-D) Mast cells (MCs) stained with Toluidine Blue (1% pH 4.0) in intraperitoneal implantation, (A) in fibrous capsule (fc) adjacent to polymer (black arrow; bar = 10 μm), and in (B) cell growth (ci) within polymer (*) with mast cells (black arrow; scale bar = 10 μm). (C) Mast cells observed in intraperitoneal fibrous capsule (fc) near polymer (*), and (D) mast cells observed in intraperitoneal fibrous capsule (fc) (black arrow; scale bar = 10 μm). (E-H) Eosinophilic granular cells (EGCs) stained with Periodic Acid Schiff (PAS) in subcutaneous implant. (E) EGCs observed inflammatory focus (if) close to fibrous capsule (fc) (bar = 20 μm); (E inset) eosinophilic granular cell (EGCs) (scale bar = 10 μm). (F) EGCs (black arrow) observed in fibrous capsule (fc) close to polymer (*) (scale bar = 20 μm). (G) inflammatory infiltrated (ii) with EGCs near to a blood vessel in fibrous capsule (fc), and (H) inflammatory infiltrated (ii) in fibrous capsule close to polymer (*) (scale bar = 20 μm). (I-L) Phagocytes (F4/80 +) immunohistochemistry. (I) Phagocytes (black arrow) in fibrous capsule (fc) near polymer (*) in subcutaneous site (scale bar = 10 μm), and (J) phagocytes (black arrow) in fibrous capsule (fc) and within cell growth within polymer (*) (scale bar = 10 μm). (K) Fibrous capsule (fc) with phagocyte (black arrow) in intraperitoneal implantation (scale bar = 10 μm), and (L) phagocytes (black arrow) observed in omentum adipose tissue (scale bar = 10 μm). (M-P) Melanomacrophages phagocytes (MMs). (M) Fibrous capsule (fc) presenting melanomacrophages (black arrow) close to polymer (*) in subcutaneous site (scale bar = 10 μm). (N) Melanomacrophage phagocytes near to fibrous capsule (fc). Note the melanomacrophage inside fibrous capsule (arrowhead; scale bar = 20 μm), and (N inset) negative control stained without primary antibody (F4/80), it is possible note that fibrous capsule is not stained (bar = 20 μm). (O) Melanomacrophage in the fibrous capsule (fc) intraperitoneal implantation, and (P) melanomacrophage phagocytes near and inside fibrous capsule (fc) at the polymer (*) (O, and P; scale bar = 10 μm).

**Fig. 4:**
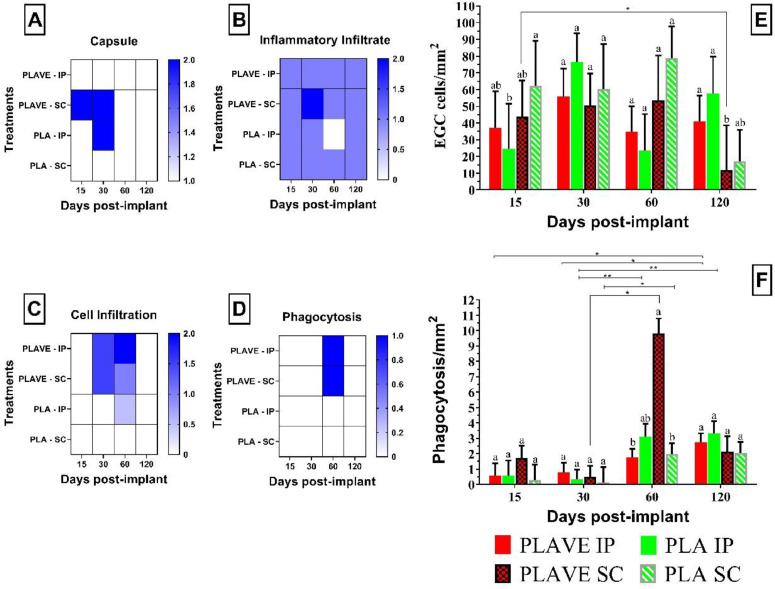
Heat map of tissue response scores of Nile tilapia (Oreochromis niloticus). The intensity of the color grade (color gradient) represents the magnitude of the score, and the more dark blue indicates a more massive tissue reaction (A, B, C, and D). The clustering tree at the days post-implantation (DPI) level was constructed at the bottom and tissue reactions were constructed at the left side, showing the relationship between time points (DPI) and histopathological analysis. The characteristic inflammatory tissue reaction to the implant was classified semi-quantitatively by scores (De Jong et al., 2005). 1- mild reaction; 2- moderate reaction; 3- intense reaction; 4- severe reaction. Hematoxylin-Eosin Staining. Means values (± SE) and Kruskal-Wallis^1^ test observed in the eosinophilic granular cells (E) and phagocytosis points counts (F) of tilapia post-implantation. Means (*n* = 7 fish/ treatment/ day sampling) followed by the same letter do not differ by the Dunn's test (*P* <0.05). Lowercase letters compare treatments in each experimental period, while symbols (* *P*<0.05; ** *P*<0.005; *** *P* ≤ 0.001). Sampling Period: 15, 30, 60, and 120 days post-implant (DPI); Two sites implantation: intraperitoneal (IP) and subcutaneous (SC); Treatments: PLA (polylactic acid), PLAVE (polylactic acid + vitamin E).

The histochemistry and immunohistochemistry analysis presented mast cells (Fig. 3A, B, C, D) and eosinophilic granular cells (Fig. 3E, F, G, H) in abundance during 15, 30, and 60 DPI, with a decrease in these cells' presence in the fibrous capsule around the polymer from 120 DPI in subcutaneous implantation (Fig. 4A). The count of these cells showed significant differences at both implantation sites throughout the experimental period, as PLA SC implantation resulted in a significant (*p*<0.05) increase in cell counts when compared to PLA IP (15 DPI). However, at 120 DPI the eosinophilic granular cell (EGC) counts in the pure PLA and PLAVE implants both implanted through the IP route showed a significant increase (*p*<0.05) in the count compared to the PLAVE SC implant. Furthermore, the evolution of the response over time revealed that EGC counts on PLAVE SC implants at 15 DPI were higher (*p*<0.05) when compared to counts performed at 120 DPI (Fig. 4E).

**Fig. 5:**
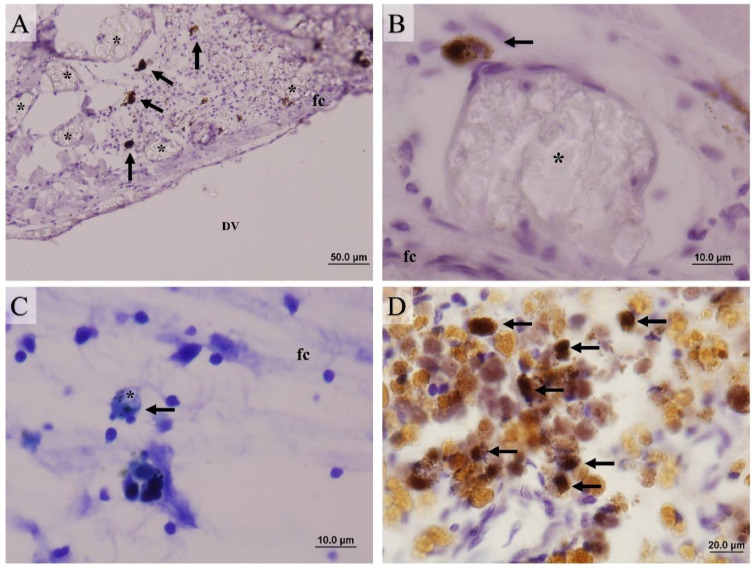
Photomicrographs of PLAVE and PLA intraperitoneal or subcutaneous implants in Nile tilapia (Oreochromis niloticus). (A) Phagocytosis sites (black arrow) stained with primary antibody (F4/80+) between fibrous capsule (fc) and polymer (DV) in subcutaneous at 60 DPI (bar = 50 µm). (B) Phagocyte cell (black arrow) stained with primary antibody (F4/80+) around piece of polymer (*) in process to phagocytosis in the fibrous capsule (fc) in subcutaneous at 60 DPI (bar = 10 μm). (C) Piece of polymer inside phagocyte cell (black arrow) stained toluidine blue (1%, pH 4.0) in intraperitoneal at 120 DPI (bar = 10 μm). (D) Phagocyte cells inside of melanomacrophage center in spleen (black arrows) stained with primary antibody (F4/80+) at 120 DPI (bar = 10 μm).

At 60 DPI it was observed in PLAVE SC implanted fish a significant (*p*<0.05) increase in phagocytosis points than other groups (PLA IP, PLAVE IP, and PLA SC) in the same period, and when compared at trial period 30 DPI (Fig. 4F). PLA IP and SC implants show the same characteristic at 60 DPI when compared to other periods. At 120 DPI the fish receiving the PLAVE IP showed significantly higher (*p*<0.05) number of phagocytic foci than at 15, and 30 DPI. (Fig. 4F). In phagocytic cells were observed biomaterial pieces inside vacuoles and pigmented aggregates in the cytoplasm. At 120 DPI, it was possible to detect F4/80+ phagocytes in tissue projections (Fig. 3I, J, K, L), in addition to observing the accumulation of melanomacrophages in the pericapsular region (Fig. 3M, N, O, P). In addition, small pieces and aggregates of the biodegrading polymer were observed in the fibrous capsule and adjacent tissue detached from the device surrounded by melanomacrophages, phagocytes, and mast cells (Fig. 5A, B, C).

Phagocytic cells (F4/80+) were observed into phagocytosis sites (Fig. 5A), very near to biopolymers at 60 DPI (Fig. 5B). These cells were observed also in spleen melanomacrophages (Fig. 5D). Besides, piece of polymer was observed inside the phagocyte cells at 120 DPI (Fig. 5C).

### 3.2 Spleen melanomacrophage centers (MMCs)

**Fig. 6:**
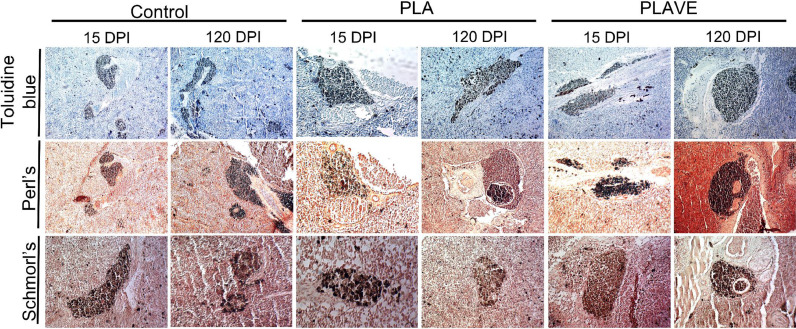
Photomicrographs stained by toluidine blue, Perl's, and Schmorl's of spleen melanomacrophages centers in Nile tilapia (Oreochromis nilotica) at 15 and 120 DPI. Control (without implant), PLA (polylactic acid) and PLAVE (polylactic acid + vitamin E) (bar = 20 μm).

PLA and PLAVE implants did not result in significant changes (*p* ≥ 0.05) in the percentage of occupation, number and area of MMCs in the tilapia's splenic tissues, during all the periods analyzed when compared to the non-implanted control fish (Table 1, Fig. 6). However, PLAVE implanted tilapia presented significant increase (*p*<0,05) in hemosiderin (15 DPI) and lipofuscin (120 DPI) pigments presented in spleen MMCs when compared to fish with PLA implants and control (Table 1). At 15 DPI, a significant increase in the amount of melanin was observed in MMCs of PLAVE tilapias compared to fish implanted with PLA, while fish implanted with PLA showed significant increase (*p* <0.05) in melanin content (120 DPI) in relation to the amounts observed in the control animals (Table 1).

**Table 1:** Means^1^ values observed in the histopathological study of spleen melanomacrophage centers of tilapia post-implantation.

Period^2^	Treatment^2^	Area^3^		Number^3^		% of occupation^3^		Melanin (%)		Hemosiderin (%)		Lipofucsin (%)	
	PLAVE	8289.72	^Aa^	3.03	^Aa^	8.43	^Aa^	0.55	^Aa^	0.29	^Aa^	0.17	^Ab^
15	PLA	4402.20	^Aa^	3.11	^Aa^	4.71	^Aa^	0.11	^Bb^	0.02	^Bb^	0.14	^Aa^
	Control	6551.73	^Aa^	4.20	^Aa^	7.00	^Aa^	0.26	^ABa^	0.01	^Ba^	0.19	^Aa^

	PLAVE	8013.81	^Aa^	3.02	^Aa^	8.45	^Aa^	0.53	^Aa^	0.34	^Aa^	0.22	^Aab^
30	PLA	7168.81	^Aa^	3.07	^Aa^	7.56	^Aa^	0.42	^Aab^	0.31	^Aa^	0.12	^Aa^
	Control	5567.34	^Aa^	2.87	^Aa^	5.67	^Aa^	0.24	^Aa^	0.31	^Aa^	0.08	^Aa^

	PLAVE	7901.05	^Aa^	2.91	^Aa^	8.14	^Aa^	0.21	^Aa^	0.73	^Aa^	0.24	^Aab^
60	PLA	8611.94	^Aa^	2.51	^Aa^	9.00	^Aa^	0.49	^Aab^	0.28	^Aa^	0.27	^Aa^
	Control	3281.91	^Aa^	3.60	^Aa^	3.33	^Aa^	0.34	^Aa^	0.17	^Aa^	0.10	^Aa^

	PLAVE	4195.28	^Aa^	3.39	^Aa^	4.62	^Aa^	0.34	^ABa^	0.43	^Aa^	0.45	^Aa^
120	PLA	3459.71	^Aa^	3.60	^Aa^	3.76	^Aa^	0.55	^Aa^	0.16	^Aab^	0.14	^Ba^
	Control	5502.49	^Aa^	3.36	^Aa^	5.83	^Aa^	0.15	^Ba^	0.18	^Aa^	0.12	^Ba^

Value of P^4^		0.198		0.463		0.139		< 0.001		< 0.001		0.004	

1 Means (*n* = 35, 7 fish X 5 fields) followed by the same letter do not differ by the Dunn's test (*P* <0.05). Capital letters in the columns compare treatments in each experimental period, while lowercase letters evaluate the evolution of each treatment among experimental periods.

2 Sampling Period: 15, 30, 60, and 120 days post-implant (DPI); Treatments: PLA (polylactic acid), PLAVE (polylactic acid + vitamin E) and control (without implant).

3 Area of MMCs (μm^2^); Number of MMCs counted by field;% of occupation of field area; Pigments present in the MMCs (Melanin, hemosiderin, and lipofuscin).

4 P value by Kruskal-Wallis test.

### 3.3 Hematological analysis

**Fig. 7:**
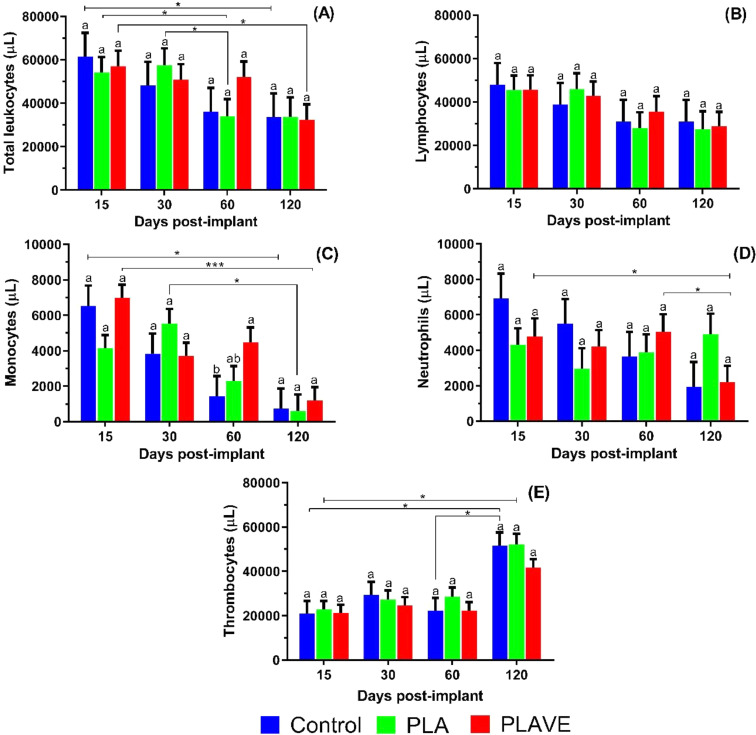
Means values (± SE) and Kruskal-Wallis^1^ observed in the white blood cell counts of tilapia post-implantation. Means (*n* = 7 fish/ treatment/ day sampling) followed by the same letter do not differ by the Dunn's test (*P* <0.05). Lowercase letters compare treatments in each experimental period, while symbols (* *P*<0.05; ** *P*<0.005; *** *P* ≤ 0.001). Sampling Period: 15, 30, 60, and 120 days post-implant (DPI); Treatments: PLA (polylactic acid), PLAVE (polylactic acid + vitamin E) and control (without implant).

The study of white blood cells revealed significant (*p*<0.05) changes between implanted fish and controls (Fig. 7). However, it was observed a significant decrease (*p*<0.05) in the number of leukocytes was observed, marked by the decrease in neutrophil and monocyte counts, which occurred gradually between the experimental days (15 to 120 DPI). Such results were accompanied by a significant increase (*p*<0.05) in the number of thrombocytes (Fig. 7). PLAVE implanted tilapia presented significant (*p*<0.05) decrease in total leukocyte counts at 120 DPI while with the control fish and implanted with PLA, this decrease (*p*<0.05) occurred at 60 DPI (Fig. 7). Tilapia implanted with PLA showed no significant (*p*>0.05) decrease in monocyte counts when compared to fish with PLAVE implants and control fish (15 DPI). However, with the evolution of foreign body inflammatory reaction 60 DPI, tilapia implanted with PLAVE showed a significant (*p*<0.05) increase in monocyte counts in relation to non-implanted control fish (Fig. 7). Non-implanted tilapia and implanted with PLAVE showed a significant decrease (*p*<0.05) in the number of neutrophils at 120 DPI (Fig. 7).

The correlation analysis between the white blood cell counts and the respiratory burst showed 72.73% (*p* = 0.0074) of negative correlation between the number of circulating leukocytes and ROS production in control animals, as well as tilapia implanted with PLAVE, showed 52.66% (*p* = 0.0040) of positive correlation between thrombocyte counts and ROS production (Table 2).

**Table 2:** Correlation analysis between respiratory burst activity values and the absolute number of leukocytes and thrombocytes counts in the blood of tilapias post-implantation.

Correlated Parameters^1^	Experimental Sampling^2^	Correlation analysis	
		ρ^3^	Prob > |ρ|^3^
	Control	−0.7273	0.0074
Leukocytes X Burst	PolA	−0.3258	0.1293
	PolAVE	−0.2729	0.1600

	Control	0.2301	0.4705
Thrombocytes X Burst	PolA	0.2284	0.2946
	PolAVE	0.5266	0.0040

1 Leukocytes= absolute number of leukocytes and thrombocytes counts in the blood; Respiratory burst activity (NBT assays).

2 Correlation among fish within each treatment. Control without implant (*n* = 28),PLA (polylactic acid) (*n* = 28), PLAVE (polylactic acid + vitamin E) (*n* = 28).

3 ρ = Coefficient of Spearman Correlation; Prob.> |ρ| – Significance Probability of ρ value.

The erythrogram study showed no significant difference between implanted fish and controls, except for an increase in hemoglobin concentrations in fish implanted with PLAVE 120 DPI (Table 3). In the evaluation between the experimental periods, there was observed a significant increase (*p*<0,05) in erythrocyte counts and hemoglobin concentrations, such findings were accompanied by a significant decrease (*p*<0,05) in mean corpuscular volume in fish implanted with PLA (120 DPI) (Table 3).

**Table 3:** Means^1^ values observed in the blood analysis of tilapia post-implantation.

Períod^2^	Treatment^2^	Erythrocytes (x10^6^/mm^3^)		Hematocrit (%)		Hemoglobin (g/dL)		MCV^3^ (fL)		MCH^3^ (pg)		MCHC^3^ (g/dL)	
	PLAVE	1.69	^Ab^	21.00	^Ab^	6.12	^Ab^	125.76	^Aa^	36.10	^Aa^	29.23	^Aab^
15	PLA	1.67	^Aab^	23.57	^Aa^	6.04	^Aab^	140.09	^Aa^	36.00	^Aa^	25.90	^Aa^
	Control	1.46	^Aa^	23.33	^Aa^	5.20	^Aa^	159.80	^Aa^	35.61	^Aa^	22.62	^Aa^

	PLAVE	1.87	^Aab^	22.86	^Ab^	6.80	^Aab^	113.59	^Aa^	36.35	^Aa^	32.89	^Aa^
30	PLA	1.64	^Ab^	24.87	^Aa^	5.87	^Ab^	138.99	^Aab^	36.25	^Aa^	26.93	^Aa^
	Control	2.07	^Aa^	25.00	^Aa^	7.61	^Aa^	124.84	^Aa^	36.68	^Aa^	35.00	^Aa^

	PLAVE	1.76	^Aab^	24.71	^Aab^	6.64	^Ab^	133.76	^Aa^	35.21	^ABa^	26.78	^Ab^
60	PLA	1.82	^Aab^	24.00	^Aa^	6.28	^Aab^	133.56	^Aab^	33.88	^Bb^	24.49	^Aa^
	Control	1.86	^Aa^	29.67	^Aa^	7.77	^Aa^	161.05	^Aa^	42.07	^Aa^	26.22	^Aa^

	PLAVE	2.55	^Aa^	32.14	^Aa^	9.44	^Aa^	111.00	^Aa^	35.63	^Aa^	28.44	^Aab^
120	PLA	2.83	^Aa^	27.08	^Aa^	8.46	^ABa^	100.96	^Ab^	31.84	^Aab^	30.75	^Aa^
	Control	2.29	^Aa^	23.33	^Aa^	7.16	^Ba^	102.83	^Aa^	32.21	^Aa^	31.25	^Aa^

Value of P^4^		0.007		0.003		< 0.001		0.002		0.027		0.016	

1 Means (*n* = 7 fish) followed by the same letter do not differ by the Dunn's-test (*P* <0.05). Capital letters in the columns compare treatments in each experimental period, while lowercase letters evaluate the evolution of each treatment among experimental periods.

2 Sampling Period: 15, 30, 60, and 120 days post-implant (DPI); Treatments: PolA (polylactic acid), PolAVE (polylactic acid + vitamin E) and control (without implant).

3 MCV-Mean corpuscular volume; MCH-Mean corpuscular hemoglobin; MCHC-Mean corpuscular hemoglobin.

4 P value by Kruskal-Wallis test.

### 3.4 Serum biochemical analyzes

**Fig. 8:**
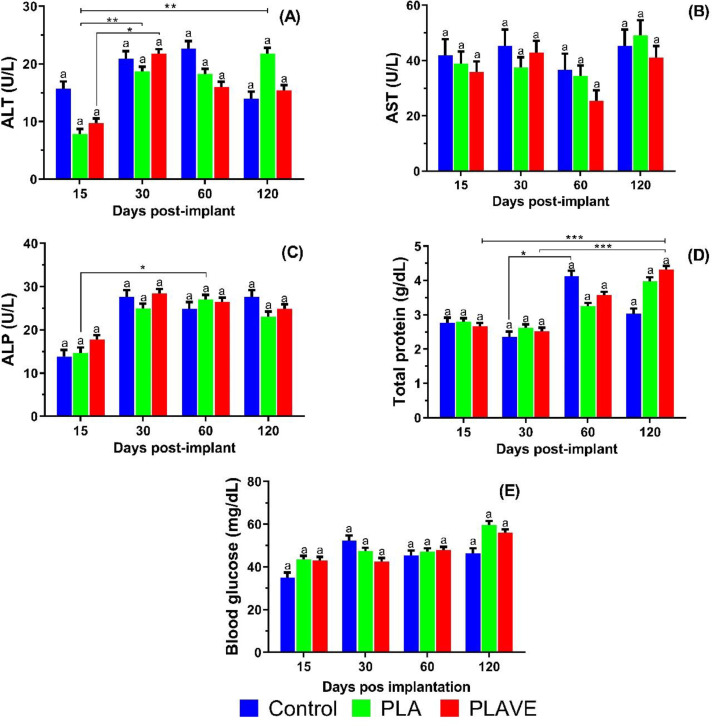
Means values (± SE) and Kruskal-Wallis^1^ observed in serum biochemical analyzes of ALT (alanine aminotransferase), AST (aspartate aminotransferase), ALP (alkaline phosphatase), total protein, and blood glucose of tilapia post-implantation. Means (*n* = 7 fish/ treatment/ day sampling) followed by the same letter do not differ by the Dunn's test (*P* <0.05). Lowercase letters compare treatments in each experimental period, while symbols (* *P*<0.05; ** *P*<0.005; *** *P* ≤ 0.001) evaluate the evolution of each treatment among experimental periods. Sampling Period: 15, 30, 60, and 120 days post-implant (DPI); Treatments: PLA (polylactic acid), PLAVE (polylactic acid + vitamin E) and control (without implant).

In the serum biochemical study (Fig. 8), the enzymatic activity did not show significant changes (*p* ≥ 0.05) among treatments. Implanted fish with PLAVE showed a significant increase (*p* <0.05) in ALT (30 DPI) and total protein (120 DPI) during the foreign body reaction (Fig 8A; 8D). In fish implanted with PLA significant increase (*p* <0.05) in ALT (120 DPI) and ALP (60 DPI) were observed (Fig 8A; 8C). Control fish (non-implanted) showed increase (*p* <0.05) in total protein levels at 60 DPI (Fig 8D).

## 4 Discussion

This study was a pioneer in evaluating the biocompatibility (clinical safety) and biodegradability of poly (lactic acid) (PLA) devices with or without vitamin E implanted subcutaneously and intraperitoneally in Nile tilapia. Since the histopathological study of polymer degradation provides important knowledges in the foreign body reaction evaluation. The determination of hematological and biochemical parameters in the clinical routine is essential, as they provide important information about the prognosis of morbid conditions [31] and allows us to assess whether the device implantation was capable of producing harmful effects to fish.

The interaction of the biomaterial's surface with the tissues triggers the onset of the inflammatory response, and tissue repair culminates in completion of this process [7]. Infiltration of mononuclear inflammatory cells and neovascularization were observed in the subcutaneous tissues and omentum of tilapia at 15 DPI. Fish implanted with polymer containing vitamin E showed more intense cellular responses and earlier capsular formation, mainly in implants present in the subcutaneous tissue, corroborating the findings of Belo et al. [24] who observed a significant increase in the accumulation of macrophages and giant cell formation in glass coverslips implanted in the subcutaneous tissue of pacus, Piaractus mesopotamicus, when supplemented with vitamin E.

The presence of eosinophilic granular cells (EGCs) in both sites of implantation (IP and SC) is related to persistent inflammatory reactions, and this response has been reported as a characteristic for the development of fibrous tissue in bony fish [37]. For Matsuyama and Iida [38], the EGC degranulation is associated with the migration of neutrophils to inflammation sites in Nile tilapia. In addition, tilapia belongs to the order of perciforms, an evolutionarily advanced class in which Mast cells / EGCs produce histamine, an important chemotactic agent for macrophages [39].

According to Mayer et al. [40], macrophages play a pivotal role in the foreign body reaction by favoring the pro-inflammatory microenvironment around the device, modulating pro-fibrotic growth factors such as TGF-β. Macrophages present in the inflamed focus are derived from circulating monocytes, and their accumulation in the device depends on the renewal rate [41]. Tilapia implanted with PLAVE showed an increase in the number of circulating monocytes at 60 DPI, suggesting the hypothesis of vitamin E participation in the kinetics of these cells between blood compartment and inflamed site.

The acute inflammatory response is triggered by mast cell degranulation, increasing vascular permeability attracting monocytes, macrophages and neutrophils inducing the release of ROS [42]. The acute inflammatory response quickly evolves to a chronic response composed of mononuclear cells in less than fifteen days [11]. An adverse effect induced by implantation of biomaterial is the exhaustion and depletion of oxidative resources of granulocytes and neutrophils, due to continuous release [13,43].

Fibroblasts and thrombocytes also participate in synthesis of granulation tissue synthesis which will support the capsule formation. Tilapia implanted with PLAVE showed a correlation between the increase in reactive oxygen species (ROS) production and the increase in the number of thrombocytes, suggesting the activation of these cells by this tocopherol and their effective participation in the immune mechanism of tilapia during chronic inflammation. However, little is known about the role of these cells in the pathophysiology of the foreign body inflammatory reaction. Thrombocytes express MHCI and MHCII, being able to process intracellular antigens and present them to effector cells, in addition to producing immunoregulatory cytokines and chemokines such as interleukin 1β [44], [45], [46]. High counts of thrombocytes have been described in the exudate during acute phase inflammation by different kinds of stimuli [47], [48], [49], [50], [51].

In the later stage of the tilapia's inflammatory reaction, there was an increase in cellular infiltration internally to the polymer and the occurrence of phagocytosis, these events were potentiated by the use of vitamin E. According to Anderson et al. [11] and Franz et al. [13], macrophages activated on the biomaterial surface express IL-1β which is chemoattractive to leukocytes in fish, stimulating the migration and accumulation of inflammatory cells, in addition to favoring phagocytic activity [52]. The increase in monocyte and neutrophil counts in SC-PLAVE-implanted tilapia (60 DPI) occurred concomitantly with the increase in cellular infiltration and phagocytosis in the polymer, highlighting the importance of this tocopherol in the activation of inflammatory cells. Dawood et al. [26] fed Nile tilapia with supplemented diets containing vitamin E nanoparticles for 8 weeks, resulting in increased gene expression for IL-1β in splenic and liver cells, and improvements in phagocytic indices. On the other hand, non-implanted tilapia showed a negative correlation in ROS production and leukocyte counts, confirming the hypothesis that in fish without inflammatory stimulus their blood leukocytes were not activated.

The decrease in neovascularization observed after 30 DPI is related to the resolution phase of the healing process [53,54]. However, the presence of giant foreign body type cells increased in the inflamed focus 60 and 120 DPI. However, the presence of phagocytic cells (F4/80+) was observed in the inflamed sites in both implantations (IP and SC). The role of these cells in the evolution of the chronic inflammatory reaction is not completely understood. These giant cells participate in the release of pro-inflammatory agents, which favor the recruitment, accumulation, and activation of new macrophages, which act in the degradation and absorption of biomaterials [9], [10], [11], [12], [13], [14], [15], [16], [17], [18], [19], [20], [21], [22], [23], [24], [25], [26], [27], [28], [29], [30], [31], [32], [33], [34], [35], [36], [37], [38], [39], [40], [41], [42], [43], [44], [45], [46], [47], [48], [49], [50], [51], [52], [53], [54]. Phagocytic cells (F4 / 80 +) have been reported in medaka fish used as a model for inflammation and oxidative stress, describing that positive cells are activated macrophages [55]. During this experiment, we did not observe the complete device biodegradation, possibly due to the short analysis period (120 days), since the time required for PLA biodegradation is estimated to be about 2 years [56].

The release of cytotoxic components damages the surrounding tissue, prolonging the inflammatory response [13]. Serious damage to tissues, infections, and foreign materials that constantly signal the activation of macrophages and stimulate the formation of melanomacrophage centers (MMCs) [57]. Interestingly, macrophages present in the tilapia's splenic melanomacrophage centers were reactive to the antibody (F4/80+). Manrique et al. [58] studied the kinetics of splenic MMCs formation in tilapia after BCG stimulation or glass coverslips implantation in the subcutaneous tissue. For these authors, the formation of MMCs in tilapia is directly related to the type of inflammatory stimulus. The application of BCG caused a significant increase in area and number of MMCs when compared to the foreign body type response. Our results of polymer implantation in tilapia corroborate the findings of these authors since there were no significant variations in the number and size of splenic MMCs during the foreign body reaction when compared to the non-implanted control fish. It has been discussed the hypothesis that MMCs are sites of humoral adaptive immune response in teleost fish, having many structural, cellular, and molecular similarities with the germinal centers in mammals [59].

There were changes in the composition of MMCs, and tilapia implanted with polymer containing vitamin E showed an increase in the amount of melanin in the initial phase at 15 DPI, while in fish implanted with pure PLA this response was late with 120 DPI, suggesting the beneficial participation of this tocopherol in the defense responses of tilapia. Classified as complex polymers, the melanin absorbs and neutralizes toxicants, cations, and free radicals, released in the catabolism of fatty acids derived from cell membrane phagocytosis [60].

Hemosiderin increase in PLAVE-implanted fish in the initial phase (15 DPI) could be associated with a better immune response, since the accumulation of this compound may be associated with the catabolism of damaged red cells and as a protective mechanism in the spleen [57]. This characteristic was observed by Manrique et al. [36] after inoculation of Nile tilapia with Aeromonas hydrophila, which causes hemorrhagic septicemia. Lipofucsin is normally observed in close association with hemosiderin granules [61], which explains its high percentage of this pigment in spleen MMCs at 120 DPI in fish implanted with PLAVE. This pigment is the result of oxidative processes and polymerization of polyunsaturated fatty acids [62], and it is associated with the uptake of red blood cells and leukocytes, effective or apoptotic [63], processes considered normal in healthy animals.

The results of blood analysis, in conjunction with histopathological studies, allow us to evaluate the safety of PLA devices for use in Nile tilapia. PLA implants resulted in decreased MCV (120 DPI) possibly due to electrolyte changes by the stress stimulus that represented the foreign body inflammatory reaction, and these findings were more significant in PLAVE implanted fish, corroborating the findings of Belo et al. [25] who verified microcytosis in vitamin E-supplemented pacus after glass coverslips implantation in the subcutaneous tissue. Vitamin E conjugated to PLA as a slow-release vehicle increased the percentage of hematocrit and hemoglobin concentration (120 DPI), confirming the results of hemosiderin present in splenic MMCs, possibly associated with a modulation of splenic hematopoietic activity. Nile tilapia supplemented with vitamin E for 10 weeks showed similar results with increased hematocrit values [64]. Despite these hematological variations observed in implanted fish, they are within the physiological limits for tilapia described by other authors [65,66].

The serum biochemical study of tilapia did not reveal changes in cytotoxicity and liver function in implanted fish, with no differences when compared to controls, except for the serum values of total protein (120 DPI). Initial decrease in ALT enzyme activity and increase in total protein, blood glucose, and alkaline phosphatase were observed throughout the study. These findings were more significant in PLAVE-implanted tilapia. Qiang et al. [27] reported that vitamin E increased the amount of total protein and decreased serum ALT activity, as noted in our study. The absence of side effects in hematological and biochemical findings, including the absence of mortality after device implantation, proves its clinical safety in Nile tilapia in the period studied.

In this context, the biocompatibility and biodegradation of polymers depend on factors intrinsic to the material itself, such as: shape, size, chemical composition, sterility, duration of contact, and degradation, in addition to external factors related to the host species and the implantation site of the device [12,67]. Therefore, the biocompatibility and biodegradation study of PLA implants in tilapia has demonstrated clinical safety and excellent evolution of foreign body inflammatory responses during the period test (i.e. 120 DPI), and these findings were significantly enhanced using vitamin E in the polymer. Our findings are promising, considering the tilapia rearing period which is eight months and the absence of mortality at 120 DPI, that is, half of the rearing period leads us to believe that the material presented here can serve as a basis for future research that seeks to study drugs or vaccines with the objective of slow and continuous release .

## CRediT authorship contribution statement

**Gabriel Conde:** Conceptualization, Methodology, Writing – original draft. **Mayumi Fernanda Aracati:** Validation, Formal analysis. **Letícia Franchin Rodrigues:** Investigation, Formal analysis. **Susana Luporini de Oliveira:** Investigation, Validation. **Camila Carlino da Costa:** Investigation, Validation. **Ives Charlie-Silva:** Methodology, Validation. **Thalles Fernando Rocha Ruiz:** Formal analysis. **Sebastião Roberto Taboga:** Formal analysis. **Marco Antonio de Andrade Belo:** Conceptualization, Supervision, Project administration, Writing – review & editing, Funding acquisition.

## Appendix Supplementary materials

Image, application 1
