# Supplementary material for: Device implant based on poly (lactic acid) with vitamin E for vaccine delivery system in Tilapia: Study for biocompatibility and biodegradation
Source: Fish Shellfish Immunol Rep. 2022 Jul 3;3:100060. doi: 10.1016/j.fsirep.2022.100060 (PMC9680062; doi:10.1016/j.fsirep.2022.100060)
Supplement: Supplementary file 1 [file mmc1.pdf]

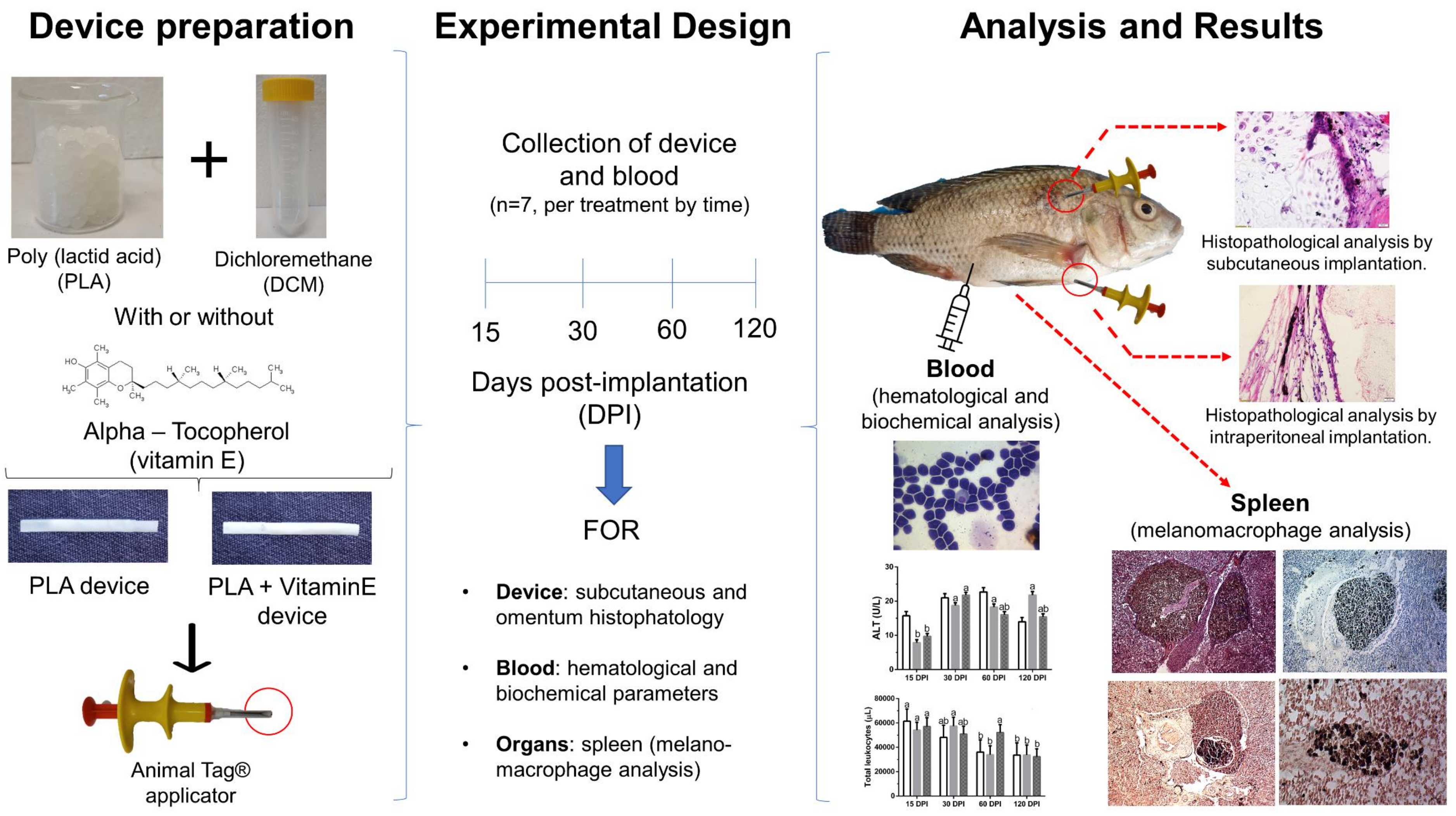
Device preparation
Experimental Design
Analysis and Results
Poly (lactid acid) (PLA)
+
Dichloremethane (DCM)
With or without
Alpha – Tocopherol (vitamin E)
PLA device
PLA + VitaminE device
Animal Tag® applicator
Collection of device and blood
(n=7, per treatment by time)
15
30
60
120
Days post-implantation (DPI)
FOR
• Device: subcutaneous and omentum histophatology
• Blood: hematological and biochemical parameters
• Organs: spleen (melano-macrophage analysis)
Histopathological analysis by subcutaneous implantation.
Histopathological analysis by intraperitoneal implantation.
Blood
(hematological and biochemical analysis)
ALT (U/L)
Total leukocytes (µL)
15 DPI
30 DPI
60 DPI
120 DPI
Spleen
(melanomacrophage analysis)
